# A multi-environment dataset consisting of official field trials for three major crops in the Nordic countries

**DOI:** 10.1038/s41597-026-07801-3

**Published:** 2026-08-24

**Authors:** Nora R. Aasen, Sybil A. Herrera-Foessel, Silius M. Vandeskog, Therése Bengtsson, Mulatu G. Dida, Isak Drozdik, Sigridur Dalmannsdottir, Egill Gautason, Jaroslaw S. Grodek, Hrannar S. Hilmarsson, Merja Högnäsbacka, Ortrud Jäck, Janne Kaseva, Antti Laine, Alex Lenkoski, Morten Lillemo, Min Lin, Anne Marthe Lundby, Shirin Mohammadi, Per J. Møllerhagen, Markku Niskanen, Rodomiro Ortiz, Maxie Skalshøi, Anna G. Þórðardóttir, Maria Thorkildsen, the NordBalFoodSec consortium, Nora R. Aasen, Sybil A. Herrera-Foessel, Silius M. Vandeskog, Therése Bengtsson, Mulatu G. Dida, Egill Gautason, Hrannar S. Hilmarsson, Alex Lenkoski, Morten Lillemo, Min Lin, Shirin Mohammadi, Rodomiro Ortiz, Anna G. Þórðardóttir, Ortrud Jäck, Lene Krusell, Charlotte D. Robertsen, Outi Manninen, Hanna Haikka, Mika Isolahti, Pernille M. Sarup, Ahmed Jahoor, Muath Alsheikh, Constantin Jensen, Helga Amdahl, Susanne Windju

**Affiliations:** 1 Norwegian Computing Center, P.O. Box 114 Blindern, 0314 Oslo, Norway; 2 Department of Plant Breeding, Swedish University of Agricultural Sciences, Lomma, Sweden; 3 Division of Food Production and Society, Norwegian Institute of Bioeconomy Research, P.O. Box 115, 1431 Ås, Norway; 4 Faculty of Agricultural Sciences, Agricultural University of Iceland, Hvanneyri, 311 Borgarbyggõ Iceland; 5 Natural Resources Institute Finland, Tietotie 4, 31600 Jokioinen, Finland; 6 Department of Plant Sciences, Norwegian University of Life Sciences, Ås, Norway; 7 SEGES Innovation, 8200 Aarhus, Denmark; 8 Sejet Plant Breeding, 8700 Horsens, Denmark; 9 Boreal Plant Breeding, 31600 Jokioinen, Finland; 10 Nordic Seed, 8464 Galten, Denmark; 11 Graminor AS, 2322 Ridabu Norway

## Abstract

Climate change will substantially impact agriculture, requiring the development of new crop cultivars adapted for resilience to future stresses. Developing a new crop cultivar is a decades-long process. Therefore climate change considerations must already be incorporated into breeding programs. National breeding programs have typically involved field trials in a single country, which has functioned satisfactorily during periods of stable climate variability. However, it is widely acknowledged that when field trial information across borders is made available, it can significantly aid plant breeding programs. This paper presents a pan-Nordic multi-environment dataset that consolidates national trial data and data from VCU (Value for Cultivation and Use) trials for spring barley, red clover, and potato, during 1970–2024. The dataset harmonizes information on yield across diverse environments, enabling comparative analyses and cross-regional assessments. By providing a unified resource for the Nordic region, this dataset supports the development of more resilient crop varieties and facilitates data-driven breeding strategies under changing climatic conditions.

## Background & Summary

Weather extremes were identified as one of the primary drivers of acute food insecurity in 2025, highlighting how climate change might threaten global food security^1,2^. To mitigate the consequences of climate change on global crop yields, new crop varieties with higher yields and better resilience to climatic stressors are needed. Plant breeding is the science of genetically improving crop cultivars with improved characteristics^3^^, [p. 4]^. This has been the main driver behind improvements in yield for the last decades^4^. However, historically it can take more than ten years from initial crossing to market release of a new crop cultivar^5^, meaning climate change considerations already need to be incorporated into breeding programs.

As part of the process of releasing new cultivars to the market, candidate varieties typically undergo 1–3 years of official trials to assess their agronomic performance and quality. These national variety trials and Value for Cultivation and Use (VCU) trials generate valuable phenotypic data that remain largely underutilized, despite their potential for cross-border data sharing and collaborative research, particularly among countries or regions with similar agro-climatic conditions^6^.

Here we present a harmonized dataset of national variety trials and official VCU trials from the five Nordic countries, Denmark, Norway, Sweden, Iceland, and Finland, focusing on three key crops for that region: spring barley (*Hordeum vulgare*), red clover (*Trifolium pratense*), and potato (*Solanum tuberosum*). The phenotypic data included in this dataset are either publicly available or were obtained upon request from the trial owners. While each country operates its own testing program through distinct institutions, there is considerable overlap in the varieties tested, and all countries share certain climatic characteristics, motivating the making of a combined dataset.

The construction of this dataset required the integration of diverse data sources, each with its own protocols for experimental design, trait measurement, and data storage. Some sources provided information on both experimental and established lines, while others were limited to specific commercial varieties. The result is a dataset that spans multiple decades and includes phenotypic observations of over 2000 varieties in almost 3000 unique year-site environments, thus offering a rich resource for studying genotype-by-environment interactions. The dataset is intended to support cross-national analyses of crop performance and environmental sensitivity across the Nordic countries. As it is constructed using data from different countries with different plot sizes, management practices and data processing strategies, its strength lies in revealing consistent, large-scale patterns across diverse environments rather than precise, trial-level analysis based on direct comparison of yield values across trials or countries.

**Fig. 1:**
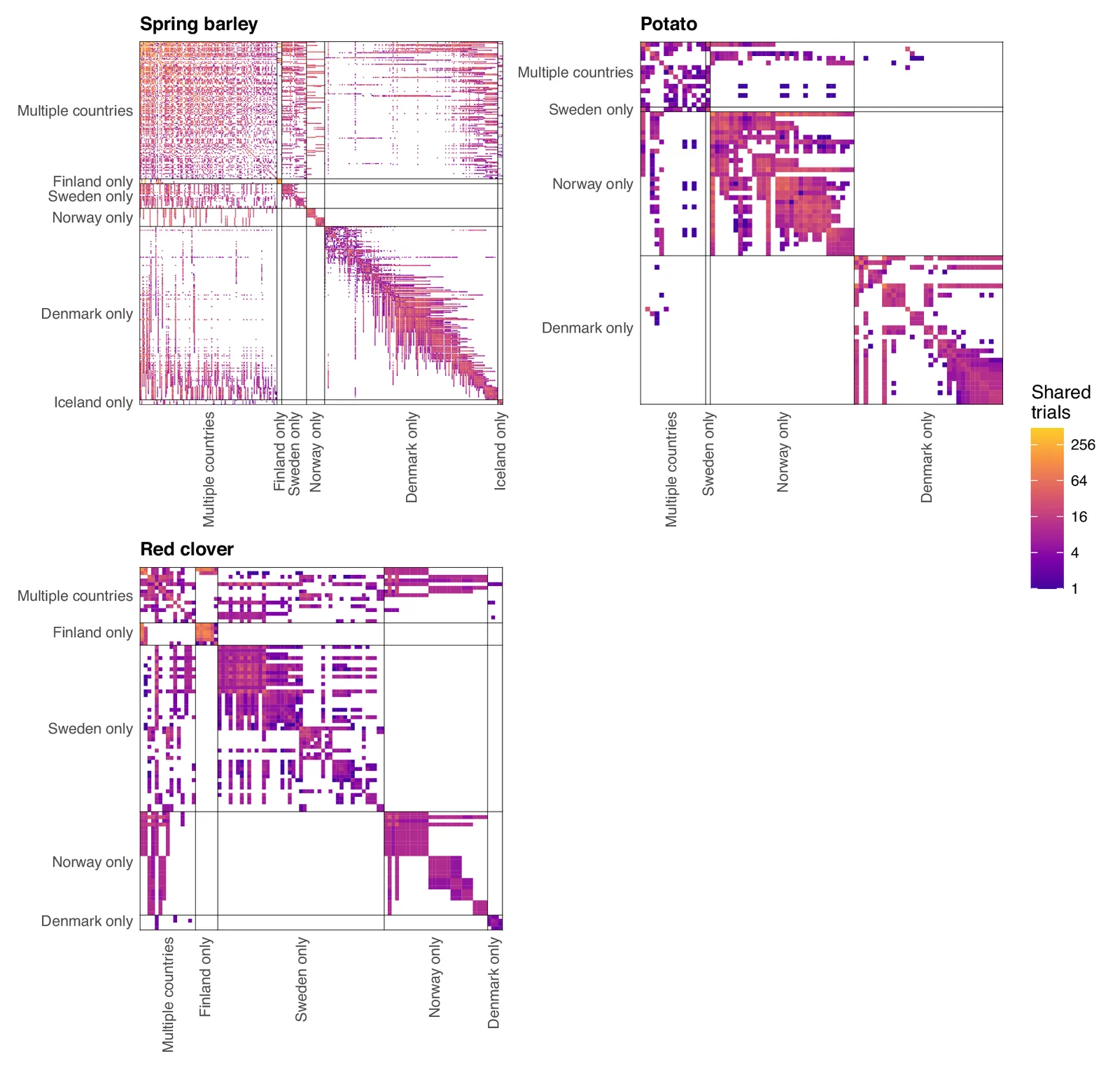
Connectivity plots for all varieties that are present in more than 30 different field trials (442, 78 and 98 spring barley, potato and red clover trials, respectively). The varieties are sorted after which countries they have been tested in. The upper leftmost parts of the connectivity plots contain varieties that are present in field trials from more than one country, while the remainder contain varieties that are only present in field trials from one of the Nordic countries. The colour of the grid cell in the *i*th row and *j*th column describes the number of field trials that contain both the *i*th and the *j*th varieties. The two varieties that appear in most trials together are KWS Irina and RGT Planet, who are present together in 304 different field trials.

Even though most of the >2000 varieties are present in field trials from only one of the Nordic countries, a considerable amount of the varieties are also tested in multiple different countries (Fig. 1). These multinational varieties are well connected with each other and with many of the varieties that only appear in field trials from one specific country. Using the multinational varieties as a type of checks may allow for strengthened inferences and to make it possible to compare different types of varieties perform over a large range of different climates and environments. However, the degree of connectivity between the multinational varieties and the varieties present in only one country varies considerably, with Sweden-only spring barley varieties being well connected to the multinational varieties, and Denmark-only potato varieties being poorly connected.

**Fig. 2:**
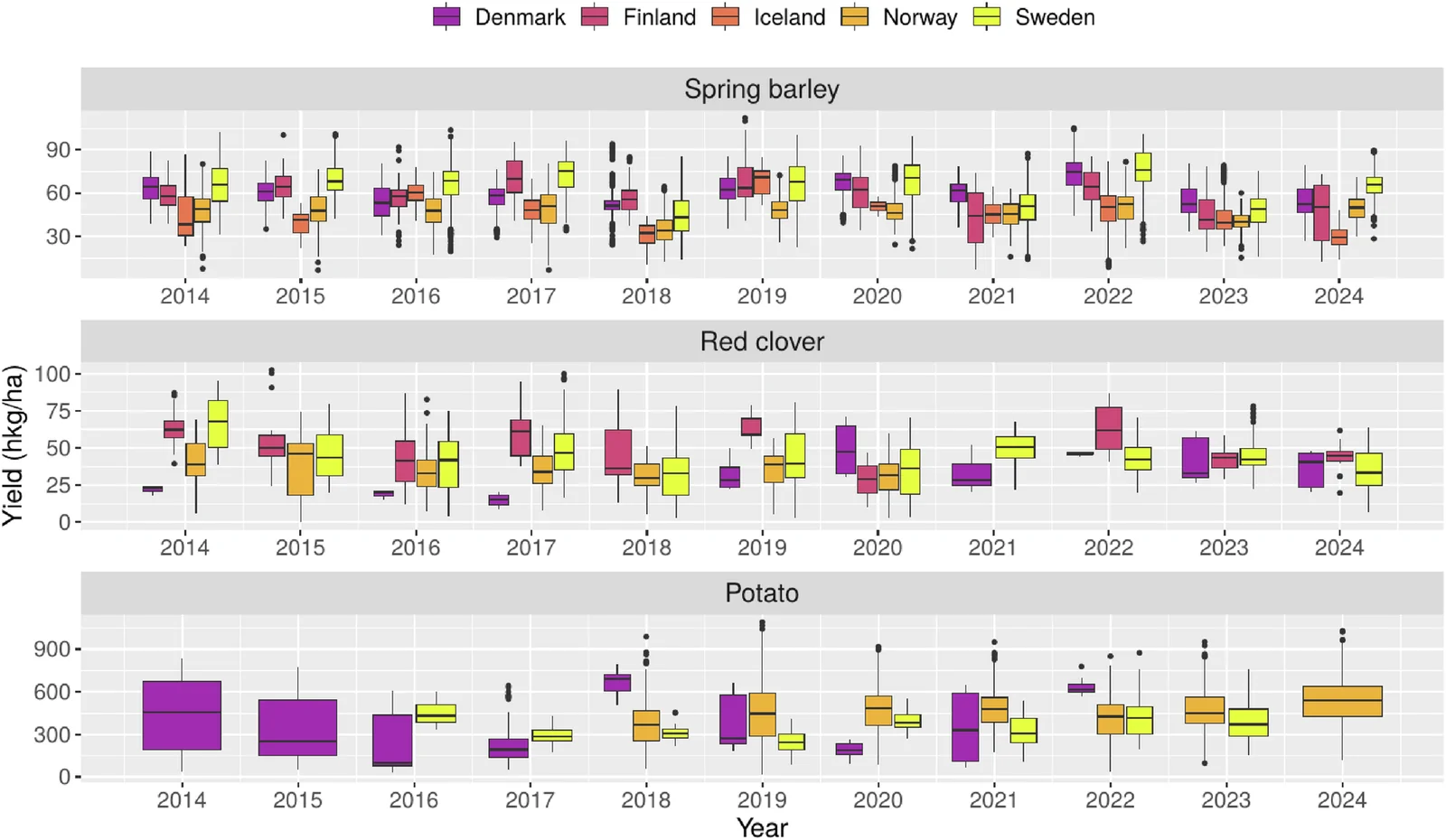
Overview of selected yield variables from the different crops, for each country and a subset of the available years (2014–2024). For spring barley we used dry matter yield, for red clover we used dry matter yield from first cut, and for potato we used (tuber) yield. Other yield variables are available for both red clover and potato. Earlier years were removed from the plots for visualisation purposes, as including yield for all years in the dataset would make them too crammed. These earlier years overall display similar means and spreads as those shown here.

**Fig. 3:**
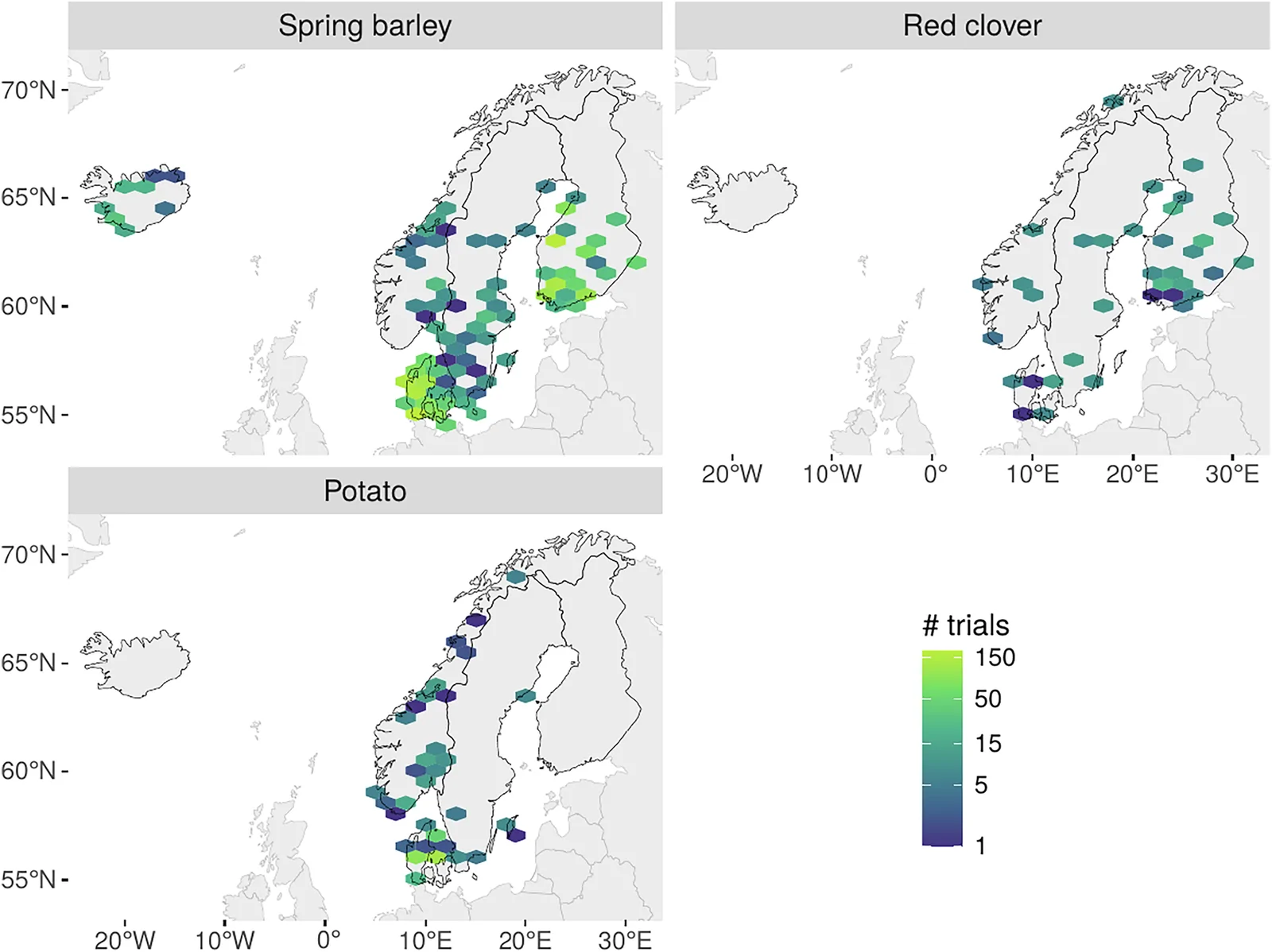
Map indicating trial sites across the Nordic countries for the three crops, with the colour of each hexagon signifying the number of trials that have been conducted inside that area.

Yield is the primary phenotypic trait reported (Fig. 2). The location of each trial is also reported (Fig. 3), thus enabling analysis of how weather variability impacts crop performance. This is particularly relevant for understanding the regional impacts of climate change and identifying both risks and opportunities for future food production^7^. Previous studies have demonstrated the utility of large-scale field trial data for predicting genotype-environment interactions using historical weather data^8,9^, and for projecting climate change impacts on different phenological traits^10–12^. Climatic adaptation patterns may extend beyond national boundaries, and harmonizing trial data across countries creates an opportunity for more comprehensive analyses of crop performance across environments, thereby supporting data-driven breeding efforts. By making this dataset openly available to breeders and agronomic researchers, we aim to support such efforts and contribute to the development of climate-resilient agriculture in the Nordic region and globally.

## Methods

### National trials and VCU trials from the Nordic countries

The collected dataset contains yield-related phenotypic traits recorded in official trials from the Nordic countries. The dataset contains both national variety trials and VCU trials. The VCU trials are performed on candidate varieties to assess their agronomic value compared to existing cultivars. The national trials are supplementary trials that give additional data on certain aspects of a crop, for instance regional adaptation or response to specific treatments.

For Norway, Finland, and Iceland we received curated data directly from the research institutes that are responsible for conducting the trials. The Danish and Swedish national variety trials and VCU trials are publicly available on a digital platform called the Nordic Field Trial System (NFTS)^13^. We downloaded all relevant data from this system.

Specific plot sizes and management practices vary between countries and years. Different countries also process their yield values in different ways, Iceland providing plot level data based on an ordinary least squares model, Finland, Sweden and Denmark providing data as best linear unbiased predictors (BLUPs), and Norway providing some data as BLUPs and some data as plot data.

Not all countries perform variety testing of all three crops, hence we only received data on spring barley from Iceland and spring barley and red clover from Finland. From Sweden, Denmark, and Norway we received data for all three crops. Following is a description of how the data from each country were collected.

#### Norway

The Norwegian trial data was collected and quality checked by the Norwegian Institute of Bioeconomy Research (NIBIO). Norwegian VCU trials are performed by NIBIO, in collaboration with the Norwegian Agricultural Advisory Service (NLR), and on behalf of the Norwegian Food Safety Authority, who funds the trials. For a plant variety (cereals, forage crops and potatoes) to be accepted on the Norwegian official list of varieties it must undergo three years of field trials. For perennial fodder crops, three planting years with three harvest years for each planting are prepared for testing.

The Norwegian data contains candidate varieties and market varieties that serve as checks. Market varieties from other countries may also appear in these trials, to assess adaptation to Norwegian climate. Some candidate varieties are eliminated after just one or two years of trials if they fail to meet the required quality standards for acceptance as a new variety. Otherwise, candidate varieties remain in the trials for three consecutive years. The data were provided as BLUPs for spring barley and plot data for red clover and potato. The Norwegian VCU trials were conducted using randomized complete block designs, with two replicates for barley and three replicates for potato and red clover.

#### Finland

The Finnish trial data was collected and quality checked by the Natural Resources Institute Finland (LUKE). Luke is responsible for coordinating official variety testing across major crops including cereals and forage legumes. The trials are performed by Luke’s local research stations, farm advisory research organization or private breeding or seed companies. Only data on checks were shared for this dataset, meaning other varieties and breeding lines in each trial were filtered out by LUKE. As a result, the Finnish contribution provides long-term environmental coverage (for red clover and spring barley) but includes a limited number of varieties per trial. The data were provided as BLUPs. Finnish VCU trials employ randomized complete block designs for experiments with small numbers of varieties and incomplete block designs, most commonly alpha-designs, for trials with larger variety sets.

#### Iceland

The Icelandic trial data was collected and quality checked by the Agricultural University of Iceland (LBHI), who are responsible for performing the Icelandic trials. For Icelandic spring barley data, the dry matter content (DMC) was estimated by weighing samples from each plot before and after drying at 60 °C for three days, which gives 95% DMC. To ensure consistency with dry matter yield reporting in the other Nordic datasets, i.e., to obtain 85% DMC, we multiplied the Icelandic dry matter yields by 1.12. Only data on varieties tested in other Nordic countries were shared by LHBI for this dataset. Before filtering, LHBI adjusted yields of the targeted varieties using an ordinary least squares model with different regression coefficients for each of the different varieties, trial numbers and for each repetition within each trial (*R*^2^ = 0.81). The data were then provided as plot data, i.e., one value for each replicate of all varieties in each trial. All the Icelandic trials were conducted using randomized complete block designs with 2–4 replicates.

#### Sweden

The field trial data for Sweden were downloaded from the NFTS website. The data were then extensively filtered and processed. More details about this are provided in the NFTS Section below.

In Sweden, VCU trials and national trials are integrated and coordinated by the Swedish University of Agricultural Sciences (SLU), as responsible for the VCU testing, and by The Rural Economy and Agricultural Societies (Hushållningssällsskapet), as responsible for the national trials. The trials are conducted at regional trial stations of both Hushållningssällskapet and SLU according to common trial instructions provided by SLU. The Swedish spring barley VCU trials changed from two-factor (with/without fungicide treatment) to a standard-treatment one-factor trial as of 2022. Additional details about the individual trials are available on the NFTS website or the webpage of SLU Fältforsk^14^. The data are provided to the NFTS website as BLUPs. The Swedish trials were either performed using randomized complete block designs or alpha-designs.

#### Denmark

The field trial data for Denmark were downloaded from the NFTS website, and went through a similar processing as the Swedish data, as described in the NFTS Section below.

In Denmark, the VCU trials, coordinated by Tystofte Fonden, and the national trials, owned by SEGES Innovation P/S (SEGES), the Danish agricultural innovation and advisory organization, are run in parallel. To increase datasets for certain varieties, including spring barley and red clover, there are some “integrated trials” where VCU varieties are also sown in national trials and vice versa. For potato, there are only national trials in the NFTS database. Some national trials evaluate for yield response to pesticide treatments, with only certain registered VCU varieties taking part. Trial plans are curated together by SEGES Innovation and Tystofte Fonden, and management is conducted by trained staff across the country. Additional details about the individual trials are available on the NFTS webpage^13^. The data are provided to the NFTS website as BLUPs. The Danish trials were either performed using randomized complete block designs or alpha-designs.

#### NFTS

NFTS publish a large collection of openly available field trial data from Sweden and Denmark at https://nfts.dlbr.dk/Forms/Forside.aspx. While the dataset is extensive, there is considerable variation in how the data are structured for different crops, years, and countries. Some of the data fields are also based on free-text entries, which have resulted in minor inconsistencies between data from different trials. Extensive filtering and processing of the available data was therefore necessary.

In the NFTS system, each field trial is given a unique 5-digit numeric ID. The trial data for ID XXXXX is available in HTML format, at the two URLs https://nfts.dlbr.dk/Forms/Dokumentation.aspx?KardexID=XXXXX and https://nfts.dlbr.dk/Forms/KomprimeretDokumentation.aspx?KardexID=XXXXX, which contain varying numbers of HTML tables and text elements for each trial. The trial data are available in both Swedish, Danish and English. The different languages can be accessed by appending &applLangID = sv, &applLangID = da or &applLangID = en to the URLs above, respectively. Different language settings provide inconsistent data for some of the trials. We therefore downloaded all Swedish trials using Swedish language settings, and vice versa for the Danish trials.

To extract as much data as possible, we examined the available NFTS web pages for all possible 5-digit IDs. We then downloaded the relevant HTML files for all IDs containing any potato, clover or spring barley data. Each NFTS trial contains 1–3 different treatment factors that are varied between different plots in the trial. We focused on either one-factor trials with variety as the treatment factor, or two-factor trials with variety and fungicide treatment as treatment factors.

By studying the raw HTML files, we found that it is not obvious, simply by looking at them, whether variety is one of the treatment factors in that trial. Even though the HTML files contain names or descriptions for all the different levels of a treatment, they do not always provide a description of the treatment itself. This is especially true for some of the older trials. To automatically identify trials where variety is a treatment factor, we first identified a list of actual variety names from trials that clearly stated variety as one of the treatment factors. We then looped over the remaining trials, assuming that a treatment contains different crop variety names if it contains three or more levels that are identical to already identified variety names. Originally, we located 1598 Danish trials and 592 Swedish trials in the NFTS system that had been clearly categorised as multi-variety trials. By searching for known varieties among the uncategorised factor types, we located 591 more Danish trials and 69 more Swedish trials which we assumed to be multi-variety trials.

Most of the field trial web pages, especially those from more recent years, contain a quality classification label, indicating whether the data from the trial are of acceptable quality. We filtered out 61 Swedish trials and 147 Danish trials with problematic classification labels. We did not remove trials without any classification labels. A closer inspection of the data revealed errors in the NFTS data, where some table columns that were supposed to contain different varieties instead contained different types of fertilizers or pesticides. We therefore filtered the extracted data to remove all crop varieties that were clearly not actual crop varieties. First, we looked for suspicious-looking variety names, e.g., those containing commas, substrings such as “10 g”, or varieties with names such as “no fertilizer”. Then, we removed all data from all trials containing one or more of these suspicious variety types. We also removed all NFTS multi-variety trials containing only two or fewer different varieties. This is a stricter filtering than for the Finnish data, which contains one-variety trials. However, the Finnish data are more standardized, and have passed through more thorough quality controls than the data that we downloaded from the NFTS servers. The NFTS data are less standardised than the Finnish trials, and several of the NFTS trials are less relevant for the purpose of this dataset (more on this in the Technical Validation Section) Therefore, to reduce the risk of including low quality data from the NFTS servers, we find it acceptable to remove all the downloaded one- and two-variety NFTS trials.

To illustrate why such strict filtering of the NFTS data is necessary, examples of valid variety names are “Miralix”, “Y-Y3”, and “SC C17-1937H (SSd)”, while examples of pesticides that were wrongly listed as varieties in at least one trial are “Citowett” and “Cerone”. It is not too hard to detect these pesticides in a small list of variety names. However, it is more challenging to locate them in a list of more than 1800 varieties. Yet, our chosen approach made it possible to identify and remove these pesticides, and other registrations that, upon closer inspection, are not actual varieties. Note that there might still be some erroneous varieties in the data set that we have not detected. Our approach might also have removed perfectly valid trials, e.g., due to inconsistent labelling of varieties across different trials. However, we believe that our chosen filtering method overall strikes a nice balance between including as much data as possible and as little faulty data as possible, with the strongest emphasis on removing faulty data.

A complete overview over how we downloaded, processed and filtered the NFTS data can be found in the fully reproducible code at Github: https://github.com/NorskRegnesentral/nordic-field-trial-data. The full procedure for downloading and processing the data has been discussed with NFTS representatives for both the Swedish and the Danish data, to ensure that the finalised data are of a high quality.

### Computational processing

Since the data came from different sources, all with individual formatting, different processing was required for different countries and crops, in order to standardise the data as much as possible. The recorded yields were given in different units, but this was rescaled to hkg/ha. Some trials had missing coordinates, which were added based on other available location information (i.e., municipality or address) combined with information from Kartverket^15^ and Google Maps, both by manually looking up and using the R package ggmap^16^. Information about the different varieties such as ploidy level, row, and early/late categorization was expanded beyond what was initially provided where possible. Spelling errors and obvious typos were, to the best of our knowledge, corrected.

From NFTS, spring barley trials were either two-factor or one-factor trials. These observations were categorized to the best of our ability into one-factor, two-factor with treatment or two-factor without treatment, where two-factor trials typically being variety trials with and without fungicide. The detailed description of the treatment is also available, but provided as is, meaning the information is written in either Swedish or Danish.

As red clover is a perennial cultivar, the same trial runs over multiple years. Observations originating from the same physical trial but different harvest years were classified using the plan number for the NFTS trials and a non unique trial id for the Finnish data, with the added criterion that the harvest years had to be consecutive. For the NFTS trials, information on the harvest year was available independently of this classification, whereas for Finnish data we always assumed that, per trial, the first year present in the data also was the first harvest year. For the Norwegian data, this classification across years was provided by NIBIO. Some red clover observations also had missing total dry and fresh matter yield (across cuts), but this was then calculated by simply adding up the individual dry and fresh matter yield, per cut and per variety for those trials.

For potato, the yield measurements in NFTS trials were provided as deviations from a reference value, often the first observation from each trial. We rescaled these so that all yield measurements are in absolute numbers. Additional information on whether the variety was industry potato or table potato was taken from the NFTS web page. Similarly, the early/late indicator was classified based on the NFTS experiment title. Cultivars that were classified as both early and late for different trials were indicated with 0.5. The early late indicator is not exact, and may deviate from the results from trials that have investigated earliness in potato. This information was not available for varieties that only appear in Norwegian trials.

## Data Records

The primary phenotypic trait reported across all three crops is yield (Fig. 2). The spring barley data contain dry matter yield. The red clover data contain dry and fresh matter yield, both per cut and across all cuts. The potato data contain tuber yield and dry matter yield. In addition, all three datasets contain information about the recorded trial site coordinates (latitude and longitude), harvest year, and sowing and harvest dates, where available. Variety-level metadata, including ploidy level, row type, and early/late maturity classification, is also provided where available. The potato data also contain additional phenological traits such as starch, number of tubers, and tuber size distribution, where available. For the Swedish and Danish NFTS trials, the experimental design type is recorded.

### Data storage

The data are openly available on the national research data repository^17^. The data files are ordered in three subfolders, one for each crop. Each subfolder contains the curated data for that crop as a csv file, as well as a README file with information about all the variables in the dataset.

## Data Overview

**Fig. 4:**
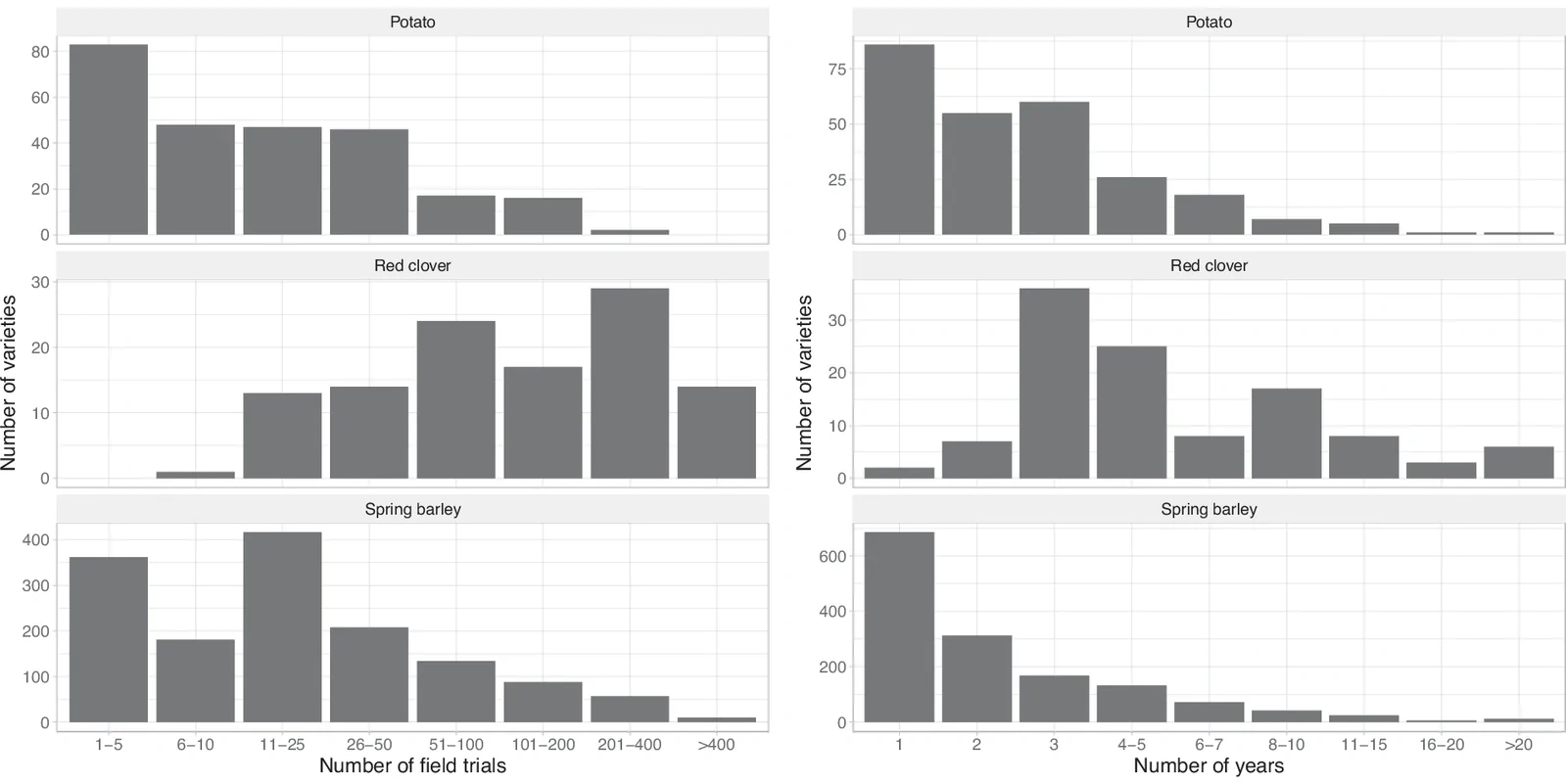
Left plot: Bar charts describing the number of field trials that each of the unique varieties are a part of. Right plot: Bar charts describing the number of years that contain field trials for each of the unique varieties.

To highlight the large variation between the data from each country, we include here a small overview of some features of the data. Key numbers that summarize the data of each crop and from each country can be found in Table 1. Furthermore, Fig. 4 provides an overview of both how many unique field trials each variety is included in, and how many years those field trials are spread across.

**Table 1:** Overview of key numbers summarizing the data from each country and in total for all three crops.

Spring barley	Total	Denmark	Finland	Iceland	Norway	Sweden
Trials	2875	1122	1065	330	132	226
Years	1970–2024	1992–2024	1970–2024	1987, 1998–2024	2014–2024	2014–2024
Sites	420	256	27	16	47	74
Environments (year × site)	2013	923	631	106	129	224
Varieties	1647	1255	25	50	78	239
Varieties per environment (mean)	19.4	26.7	5	7.6	26.2	31.6
Varieties per environment (min)	1	7	1	3	19	8
Varieties per environment (max)	118	118	10	20	36	61
**Red clover**	**Total**	**Denmark**	**Finland**		**Norway**	**Sweden**
Trials	315	14	185		44	72
Years	1975–2024	2004, 2009–2014, 2016–2017, 2019–2024	1975–2020, 2022–2024		2010–2020	2011–2024
Sites	45	7	21		6	11
Environments (year × site)	501	28	322		60	91
Varieties	129	16	10		37	66
Varieties per environment (mean)	6.7	4.2	3.9		12.6	13.3
Varieties per environment (min)	1	3	1		6	7
Varieties per environment (max)	21	9	8		19	21
**Potato**	**Total**	**Denmark**			**Norway**	**Sweden**
Trials	446	291			120	35
Years	1992–2024	1992–2022			2018–2024	2016–2023
Sites	87	37			38	12
Environments (year × site)	272	129			108	35
Varieties	274	202			42	30
Varieties per environment (mean)	8.8	8.4			10.1	6.5
Varieties per environment (min)	3	4			8	3
Varieties per environment (max)	27	27			16	9

## Technical Validation

The individual field trials were performed by agronomists and were quality-checked by the responsible national institutes before we received or downloaded the data. Our own technical validation focused on ensuring consistency across data providers and on removing NFTS data that do not fit the purpose of the dataset.

The downloaded data from NFTS contains a mixture of trials, some of which may be less relevant. This includes trials with no recorded yield values, possibly faulty trial data containing implausible factor labels (e.g., fertiliser names appearing in variety columns), test field trials with unusual setups, and other trials with unusual fertiliser or pesticide schemes that may not be comparable to regular VCU trials or national trials from other countries. Visual inspection of different summary statistics were used to detect erroneous registrations and typos of yield, location, sowing date or harvest date from the NFTS data. Examples of such errors include harvest and sowing dates from different years than the field trial year, coordinates in the ocean, or from different countries than the field trial country and field trials where only one or two of the available varieties contain non-missing yield values. Data from such field trials were either removed or corrected using other available information from the NFTS webpage, if available. Trials with missing coordinates were geolocated using other available location information (municipality, address) in combination with Kartverket^15^ and Google Maps, or removed if geolocation was not possible.

To examine consistency across data providers, we evaluated summary statistics such as the box plots in Fig. 2, to look for considerable differences in yields or yield trends between different countries. The box plots in Fig. 2 show overall yield distributions for all varieties within each crop type. However, we also examined similar plots based on only one variety at a time, for the most common varieties within each crop type. We grouped the data by different countries and by different latitudes, to look for problematic patterns, such as one variety performing well in one country and poorly in a neighbouring country, or at different latitudes. Our overall findings based on these examinations are that the yield levels, and other relevant parts of the data, appear to be broadly consistent across all countries within each crop.

## Usage Notes

As previously noted, there can be considerable variations in the field trials within each country and between different countries, i.e., differences in plot sizes, management practices, data processing strategies (e.g., providing yields values as BLUPs vs plot data), soil types, fertilisation and pesticide usage. However, all trials follow standardized national protocols intended to facilitate as reliable cultivar comparisons as possible between different locations and years within each country. Additionally, substantial efforts were made to standardize, curate, and align the datasets to ensure consistency. However, the dataset is not intended for direct comparison of absolute values across trials or countries. Instead, analyses should be based on within-trial comparisons, where genotype performance is evaluated relative to other entries tested under the same experimental conditions and statistical framework, thereby reducing the impact of methodological differences. Furthermore, the dataset is designed to support analyses focused on large-scale patterns across environments, such as genotype-by-environment interaction and climatic adaptation, where centering, scaling, or re-estimation of effects is typically applied, and where between-country and between-environment variability is substantially larger than differences arising from, e.g., using a combination of BLUPs and plot data averages. Users should consider this when deciding when and how to use the dataset.

The dataset does not contain climate or soil covariates directly. However, each trial record includes geographic coordinates and harvest year, enabling straightforward linkage to publicly available weather and climate datasets. Suitable resources for the Nordic region include E-OBS^18^ and the ERA5 reanalysis^19^. For climate projections, the EURO-CORDEX ensemble^20^ provides high-resolution regional scenarios. Soil data can be sourced from, e.g., the European Soil Data Centre (ESDAC^21^) or SoilGrids^22^.

It is a known problem that variety names are not historically unique, and may change both over time and across borders. Multiple variety names in the dataset may therefore represent the same genotype. We have not attempted to link these together, using public genotypic datasets, as this would require considerable amounts of manual work for the full dataset of almost 2000 different varieties. However, such work might be more feasible for users of the dataset that wish to focus on a smaller subset of the data.

## Data Availability

The data are openly available, under a CC BY 4.0 license, on the national research data repository DataverseNO^17^ at 10.18710/IZ3EFE, with supplementary README files describing the contents and folder structure.

## Code availability

Code used for data processing is available on GitHub (https://github.com/NorskRegnesentral/nordic-field-trial-data). The raw NFTS data can be downloaded from the website using the provided R script.
